# High-Temperature Wettability and Interactions between Y-Containing Ni-Based Alloys and Various Oxide Ceramics

**DOI:** 10.3390/ma11050749

**Published:** 2018-05-07

**Authors:** Jinpeng Li, Huarui Zhang, Ming Gao, Qingling Li, Weidong Bian, Tongxiang Tao, Hu Zhang

**Affiliations:** 1 School of Materials Science and Engineering, Beihang University, Beijing 100191, China; lijinpeng@buaa.edu.cn (J.L.); gao_ming@buaa.edu.cn (M.G.); qingling@buaa.edu.cn (Q.L.); bianweidong@buaa.edu.cn (W.B.); 2 Qingdao Institute of New Material technology of Beihang University, Qingdao 266000, China; taotongxiang@hdmater.com

**Keywords:** high temperature ceramic, high-temperature wettability, interfacial interaction, MCrAlY alloys

## Abstract

To obtain appropriate crucible materials for vacuum induction melting of MCrAlY alloys, four different oxide ceramics, including MgO, Y_2_O_3_, Al_2_O_3_, and ZrO_2_, with various microstructures were designed and characterized. The high-temperature wettability and interactions between Ni-20Co-20Cr-10Al-1.5Y alloys and oxide ceramics were studied by sessile drop experiments under vacuum. The results showed that all the systems exhibited non-wetting behavior. The contact angles were stable during the melting process of alloys and the equilibrium contact angles were 140° (MgO), 148° (Y_2_O_3_), 154° (Al_2_O_3_), and 157° (ZrO_2_), respectively. The interfacial reaction between the ceramic substrates and alloys occurred at high temperature. Though the ceramics had different microstructures, similar continuous Y_2_O_3_ reaction layer with thicknesses of about 25 μm at the alloy-ceramic interface in MgO, Al_2_O_3_, and ZrO_2_ systems formed. The average area percentage of oxides in the alloy matrices were 0.59% (MgO), 0.11% (Al_2_O_3_), 0.09% (ZrO_2_), and 0.02% (Y_2_O_3_), respectively. The alloys, after reacting with MgO ceramic, had the highest inclusion content, while those with the lowest content were in the Y_2_O_3_ system. Y_2_O_3_ ceramic was the most beneficial for vacuum induction melting of high-purity Y-containing Ni-based alloys.

## 1. Introduction

With the development of the aviation industry, the inlet temperature of military large-scale engine turbines has been increased to 1850–2000 K. The working temperature of hot-end components of aero-engines is getting higher and higher, and is affected by the oxidation and corrosion of high-temperature gas [1,2]. MCrAlY (M = Ni and/or Co) alloys are widely used as overlays or bond coats for thermal barrier coatings (TBCs) to protect gas turbine blades and other hot components against high-temperature oxidation and hot corrosion [3,4].

At present, the common methods for preparing superalloys include vacuum induction melting (VIM), vacuum arc melting (VAR), electroslag remelting (ESR), electron beam remelting (EBR), and a combination of the two or three methods [5,6]. VIM has been almost the most common method for nickel-based superalloys. However, VIM is the only vacuum melting method that uses a refractory crucible made of oxides such as SiO_2_, CaO, Al_2_O_3_, ZrO_2_, and MgO [7,8]. Unfortunately, the refractory is a contamination source of oxygen because the metal/refractory interface has a thermal mechanical infiltration and thermophysical effects, which lead to crucible disintegration [9,10]. Moreover, during the melting process, interfacial chemical reactions may occur between the ceramics and the active elements, such as Ti, Hf, Al, Cr, and so on. Reaction products precipitate at the alloy-ceramic interface and within the alloy matrix, consequently deteriorating the microstructure and mechanical properties of the resultant Ni-based alloys [11,12,13,14]. Qing [15] found that Cr, Hf levels had the effect of accelerating interface reaction between IC6, K465, K4648, K488, and K002 alloy melts and silicon oxide core, and reaction products in alloys were mainly hafnium oxides and alumina. Kanetkar et al. [16] and Valenza et al. [17] observed the phenomenon that oxides were formed on the surface of a zirconia substrate after sessile drop experiments. However, the reason for the formation of oxides was not clear and the information of wetting behavior under high temperature between them was severely deficient.

The wettability of ceramics with regard to molten alloys is of importance to understand the reaction mechanisms between the alloys and ceramics [18,19]. Previously, Jiang et al. [20] analyzed the mechanisms of the interfacial reactions between Zr-containing nickel-based superalloy and magnesia partially stabilized zirconia ceramic by means of wetting experiments. They found that the contact angle was larger than 90° during the continuous temperature rise, the equilibrium contact angle was 92°, and the interfacial reaction was influenced by the reduction of ZrO_2_. A recent work by Chen et al. [21] showed that the interface reactions between Ni_3_Al based superalloy and Al_2_O_3_ ceramic occured as C and Hf contents reached a critical value. In addition, adsorptions of Hf and interface reactions improved the wettability obviously. Kritsalis et al. [22] also reported that Ni alloys containing up to 20 at % Cr, wetting and thermodynamic adhesion to alumina were mainly determined by the concentration of oxygen dissolved in the alloys. Thus, an appropriate refractory is of importance with regard to the wetting process, as well as the interfacial reactions between the alloys and ceramics.

It is well established that an element, such as aluminum, in the alloy must be selectively oxidized to form a continuous external scale which is resistant to cracking and spalling. The adherence of the oxide scale is crucial to oxidation resistance. Some elements, such as yttrium, hafnium, and cerium (oxygen active elements), can be used to very substantially improve oxide scale adherence [23]. MCrAlY alloys contain Y at concentrations and Y improves the adherence of Al_2_O_3_ scales to these coatings. To obtain high-quality MCrAlY alloys, ceramics with appropriate materials are essential. This will contribute to controlling appropriate levels of wettability and reducing interfacial reactions with the Ni-based alloys during melting. Unfortunately, the influence of various oxide ceramics on high-temperature wettability and interactions between Y-containing Ni-based alloys and ceramics have rarely been investigated.

In this paper, four different oxide ceramics, including MgO, Y_2_O_3_, Al_2_O_3_, and ZrO_2_, are self-designed and characterized. The influence of various ceramics on the high-temperature wettability and interfacial reactions with regard to Ni-20Co-20Cr-10Al-1.5Y alloys and ceramics were studied. Meanwhile, the corresponding mechanisms were clarified.

## 2. Materials and Methods

The Ni-20Co-20Cr-10Al-1.5Y (wt %) master alloys were prepared by a 100 KW ZG125C vacuum induction furnace (Herz Special Metallurgy Plant, Shanghai, China) equipped with appropriate amounts of high-purity metals, Ni (99.98%), Co (99.50%), Cr (99%), Al (99.99%), and Y (99.9%), as raw materials. The 3 mm × 3 mm × 3 mm cubes (about 0.16 g) obtained from the central part of the master alloys were used for the wetting experiments.

The raw material particle size and some specific parameters were designed and given in Table 1. Polyvinyl alcohol (PVA) as a binder was used to prepare ceramic powder and milled for 2 h, and then dried at the controlled temperature of 298 K and the relative humidity of 30 ± 1% for 1 h. Four different oxide ceramics were prepared in dimensions of Φ 21 mm × 10 mm by the dry-pressing method using a uni-axial pressure machine (YLJ-40T, Shenyang Kejing Automation Equipment Co., Ltd., Shenyang, China) and sintered using a high-temperature box resistance furnace. The sintering temperature is 1913 K, the heating rate is 20 K/min, and furnace cooling is performed. After sintering and, consequently, before performing the contact angle experiments, the substrate surface was ground and polished in order to control the roughness at the S/L interface.

**Figure 1:**
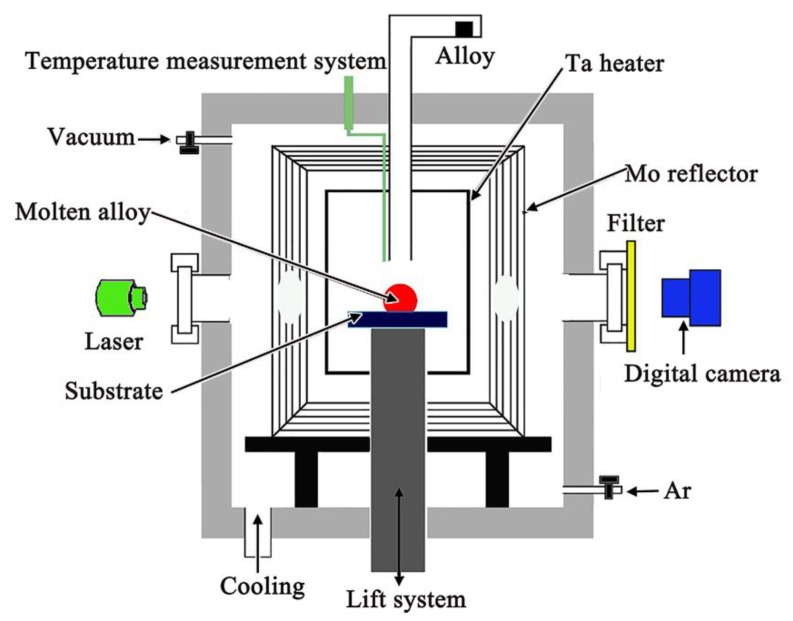
Schematic diagram of the improved sessile drop equipment.

The wetting experiments between the ceramics and alloys were performed using an improved sessile drop equipment, as shown in Figure 1, which offered a distinct advantage in measuring initial contact angles and preventing the occurrence of the interfacial reactions between the alloys and oxide ceramic substrates during heating. The whole experimental process was in a vacuum atmosphere to prevent active elements from evaporation and oxidation, and the oxygen partial pressure in this condition should be lower than 10^−8^ Pa [24]. When the temperature was 1873 K, the alloy, which was located at the top of the equipment, was dropped. The spreading process was recorded by the CCD camera. The contact angles were directly measured from the captured drop profiles using an axisymmetric-drop-shape analysis (ADSA) program with an error within ±1° [25].

After the sessile-drop experiments, the solidified samples were embedded in resin and then cut and polished for microstructural observations. The microstructure of the samples were analyzed by scanning electron microscope (SEM, JSM6010, Japan Electronics Co., Ltd., Tokyo, Japan). The chemical compositions were analyzed by energy dispersive spectrometer (EDS, Oxford INCAPentaFET-x3, Japan Electronics Co., Ltd., Tokyo, Japan). The phases of the samples were identified by X-ray microdiffraction (D8 Discover with GADDS, Bruker, Karlsruhe, Germany). The open porosity level of each as-obtained ceramic substrates was measured (according to GB/T 1966-1996). The surface line roughness (Ra) of each ceramic substrate was measured using a LEXT OLS4000 3D laser (12.5mm, performed three times, Olympus Corporation, Tokyo, Japan).

## 3. Results

### 3.1. Microstructural of Ceramic Substrates

**Figure 2:**
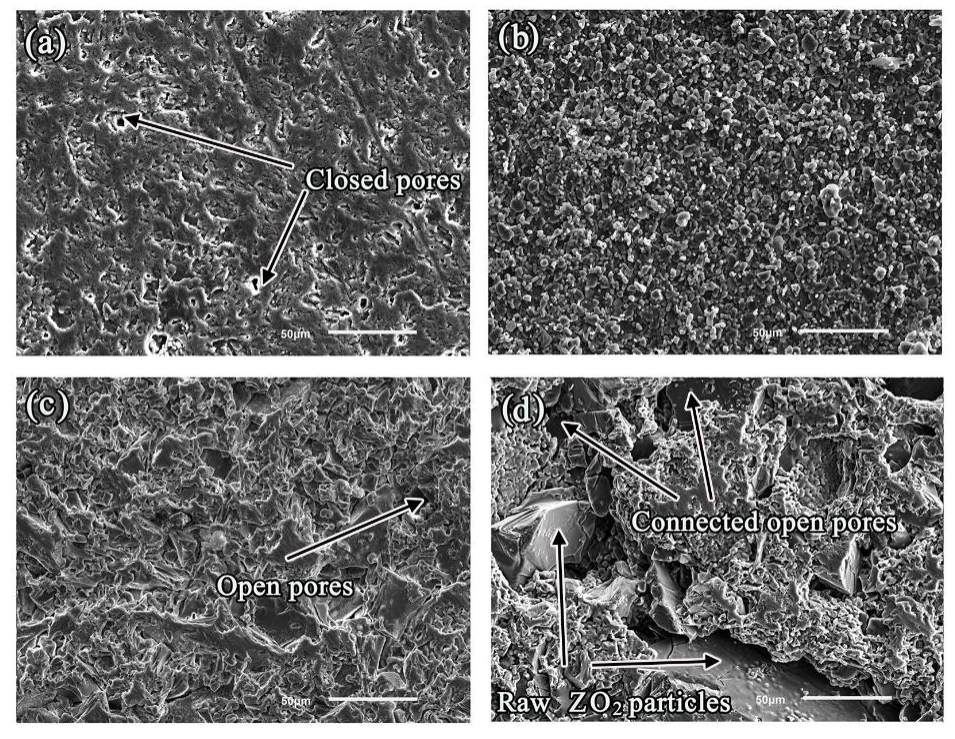
Microstructures of the various oxide ceramic substrates before sessile drop experiments: (**a**) MgO; (**b**) Y_2_O_3_; (**c**) Al_2_O_3_; and (**d**) ZrO_2_.

The microstructures of polished surface including MgO, Y_2_O_3_, Al_2_O_3_, and ZrO_2_ substrates prior to the wetting experiments are shown in Figure 2. There was a distinct difference in pores and the exhibition of ceramic particles. In the case of the MgO substrate, MgO particles were completely sintered together, and a few closed pores could be observed within the finely-sintered MgO blocks. In the case of the Y_2_O_3_ substrate, though the degree of sintering of the Y_2_O_3_ particles was relatively low, the majority of Y_2_O_3_ particles were sintered together. In the case of the Al_2_O_3_ substrate, the degree of sintering of the Al_2_O_3_ particles was relatively high and open pores were distributed independently throughout the sintered ceramic blocks. However, in the cases of the ZrO_2_ substrate, the open pores were interconnected, and some raw ZrO_2_ blocks could be observed. The key differences can be summarized as follows: the degree of sintering densification gradually increased, MgO > Al_2_O_3_ > Y_2_O_3_ > ZrO_2_.

**Figure 3:**
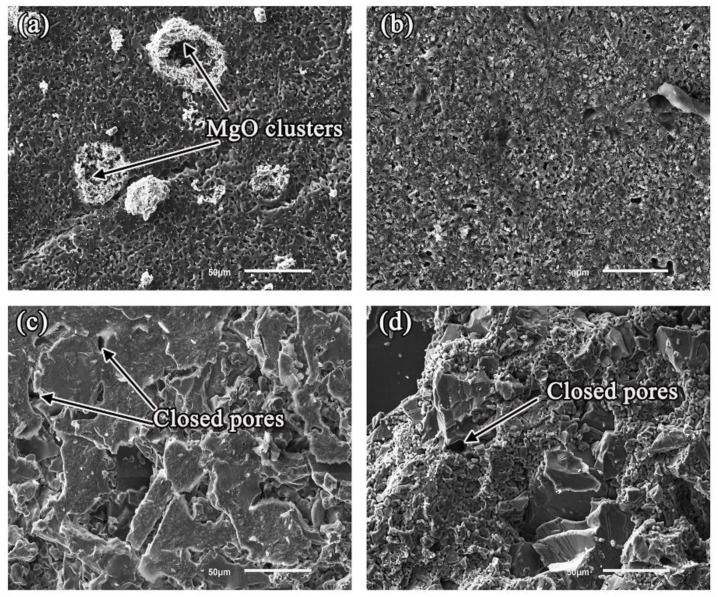
Microstructures of the various oxide ceramic substrates after wetting: (**a**) MgO; (**b**) Y_2_O_3_; (**c**) Al_2_O_3_; and (**d**) ZrO_2_.

Figure 3 shows the microstructures of untreated substrates in contact with the alloys near the three-phase line, posterior to the wetting experiments. The ceramic structures contacted with the alloy melts had a liquid-phase contact and re-sintered, and no alloy liquid was penetrated into the substrates. The four kinds of crucibles have superior corrosion resistance. In the case of the Al_2_O_3_ and ZrO_2_ systems, Al_2_O_3_ and ZrO_2_ particles re-sintered into larger ceramic blocks, and the open pores became closed pores. In the case of the MgO system, white protrusions were found and EDS analysis confirmed that the protrusions were composed of MgO clusters, which was due to the alloy melts adhering to the substrate during the wetting process, so that MgO particles on the surface of the substrate were stuck.

### 3.2. Wetting Behavior of the Alloys on the Oxide Ceramics Substrates

**Figure 4:**
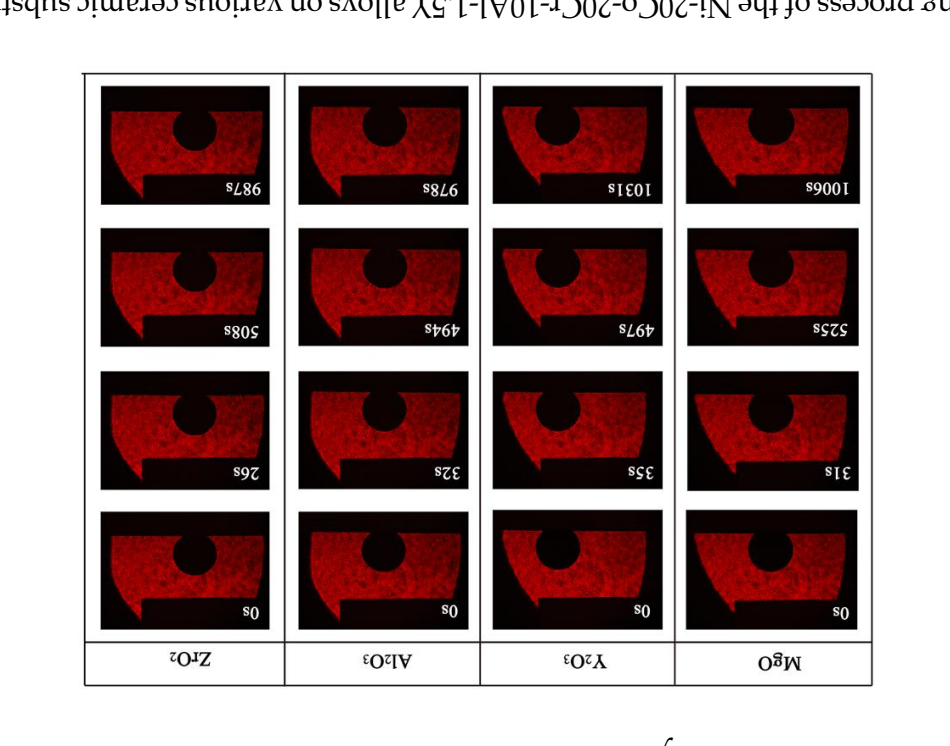
Wetting process of the Ni-20Co-20Cr-10Al-1.5Y alloys on various ceramic substrates substrates at 1873 K.

**Figure 5:**
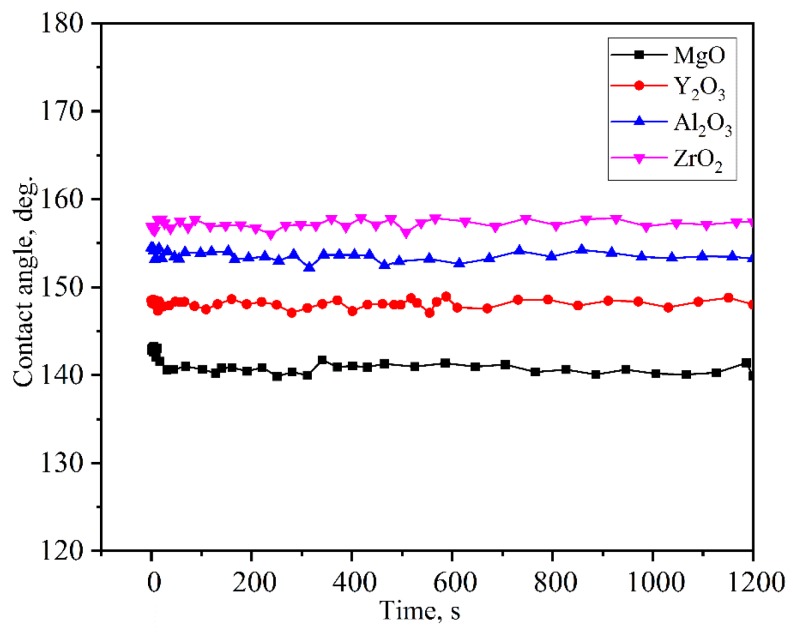
Variation in the apparent contact angle with time with regard to the Ni-20Co-20Cr-10Al-1.5Y alloys on the various oxide ceramic substrates at 1873 K.

Figure 4 and Figure 5 show the changes in contact angle of the drop during the continuous time rise, at 1873 K. With the increase of the experiment time, the contact angles of molten alloys on MgO, Y_2_O_3_, Al_2_O_3_, and ZrO_2_ substrates rapidly became stable and was larger than 140°. As evident from Figure 5, alloys had a similar wettability on all of these substrates with initial contact angles in the range of 143° to 157° and equilibrium contact angles (after 20 min) between 140° and 157°. In the case of MgO, Y_2_O_3_, Al_2_O_3_, and ZrO_2_ substrates, the equilibrium angles were 140°, 148°, 154°, and 157°, respectively. There was no characteristic transition in wettability and all the systems that exhibited wetting behavior were found to be non-wetting when the type of substrates changed. This indicated that the Ni-20Co-20Cr-10Al-1.5Y alloys and the four different oxide ceramics were non-wetting.

### 3.3. Interfacial Reactions between the Alloys and Ceramics

**Figure 6:**
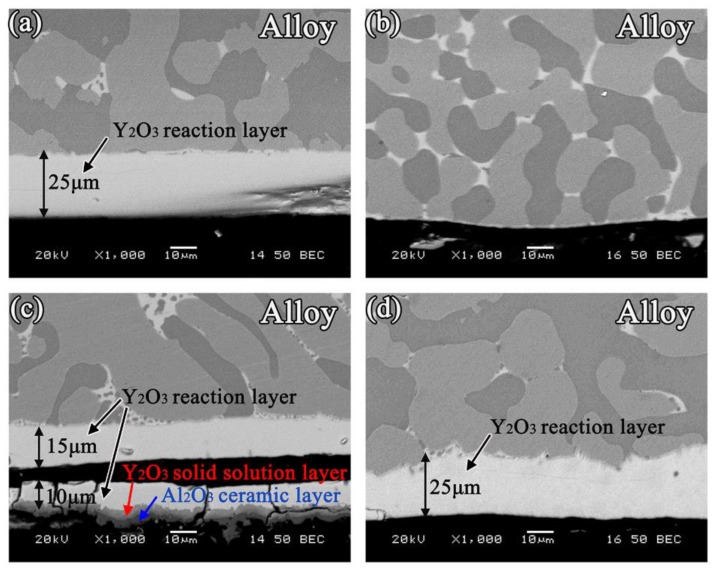
Morphologies of the metal/substrate interfaces: (**a**) MgO; (**b**) Y_2_O_3_; (**c**) Al_2_O_3_; and (**d**) ZrO_2_.

**Figure 7:**
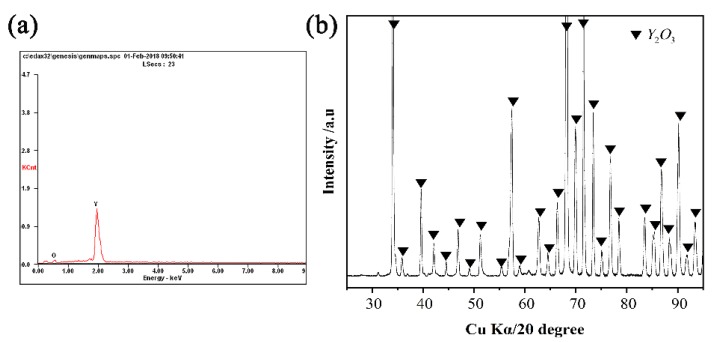
EDS and micro-XRD patterns of the metal/MgO substrate interface.

The typical SEM images of the alloy/ceramic interfaces, sectioned perpendicular to the interfaces of the four non-wetting systems, are shown in Figure 6. It can be seen that the interface was clean and there were no sand adhesion and reaction layers for the Y_2_O_3_ system (Figure 6b). However, an approximately 25 μm continuous layer of white product were observed at the interface for the MgO, Al_2_O_3_ and ZrO_2_ systems (Figure 6a,c,d). EDS analysis indicated that Y and O are the two dominant elements, which suggested that this layer mainly corresponded to Y_2_O_3_, and XRD analysis further confirmed it (Figure 7). It was indicated that interfacial reactions between MgO, Al_2_O_3_, and ZrO_2_ substrates, and Ni-20Co-20Cr-10Al-1.5Y alloys occurred at high temperature. In addition to the interface reaction layer, a sand adhesion layer was also observed at the interface of the Al_2_O_3_ system (Figure 6c). EDS analysis indicated that Al and O were the two dominant elements, which suggested that the sand adhesion layer was Al_2_O_3_ stripped from the ceramic surface. Interestingly, there was a thin layer of Y_2_O_3_ solid solution between the reaction layer and the sand adhesion layer, forming a sandwich-like structure as shown by the image of the alloy of Al_2_O_3_ system.

**Figure 8:**
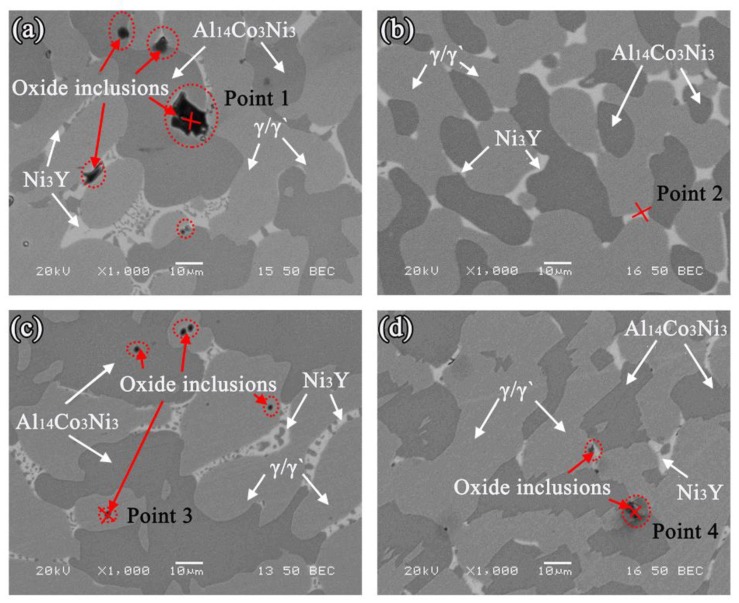
The morphologies of the alloy matrices: (**a**) MgO; (**b**) Y_2_O_3_; (**c**) Al_2_O_3_; and (**d**) ZrO_2_.

**Figure 9:**
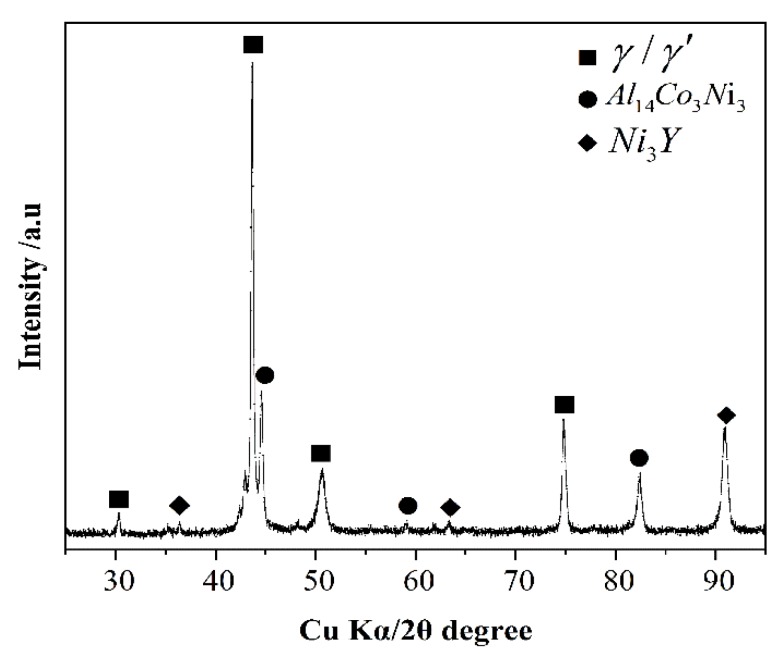
X-ray microdiffraction patterns of the alloys of the Y_2_O_3_ systems.

Images of the cross-sections of the alloys near the triple line and the alloy matrix are shown in Figure 8. The Ni-20Co-20Cr-10Al-1.5Y alloys had the same phase composition as the cast microstructure on the four different substrates, including the γ phase (fcc structural matrix phase), γ’ phase (Ni_3_Al), Al_14_Co_3_Ni_3_ phase, and Ni_3_Y phase, as shown in Figure 9. Some oxide particles were also found in the alloy matrices of all systems. The area percentages of the oxide inclusions, based on the images of the longitudinal sections, are shown in Table 2 and Table 3. The alloy of the MgO system had the greatest inclusions content, while the alloy of the Y_2_O_3_ system had the lowest content. The average area percentage of the oxide particles of the four systems were 0.59% (MgO), 0.11% (Al_2_O_3_), 0.09% (ZrO_2_), and 0.02% (Y_2_O_3_), respectively.

## 4. Discussion

### 4.1. Wetting Kinetics and Driving Force

In the four systems, the contact angles were stable during the melting process of alloys and the equilibrium contact angles were larger than 90°. All the systems exhibited non-wetting behavior. In order to try to explain the changing in the wetting characteristics as a function of both alloys and substrate composition, the surface tension and the roughness surface were carried out on all samples.

The surface tension of molten metals is the most important thermophysical property which dominates thermo and hydrodynamic processes at the surface, the surface tension value is larger, and the spread of alloy melts is more difficult [26]. The solid/liquid surface tensions for substrates were calculated by analyzing the photographs of the droplet using Young’s equation:

(1) $$\gamma_{lv}\cos\theta =\gamma_{sv}-\gamma_{sl}$$

(2) $$W_{A}=\gamma_{lv}(1+\cos\theta )$$

**Figure 10:**
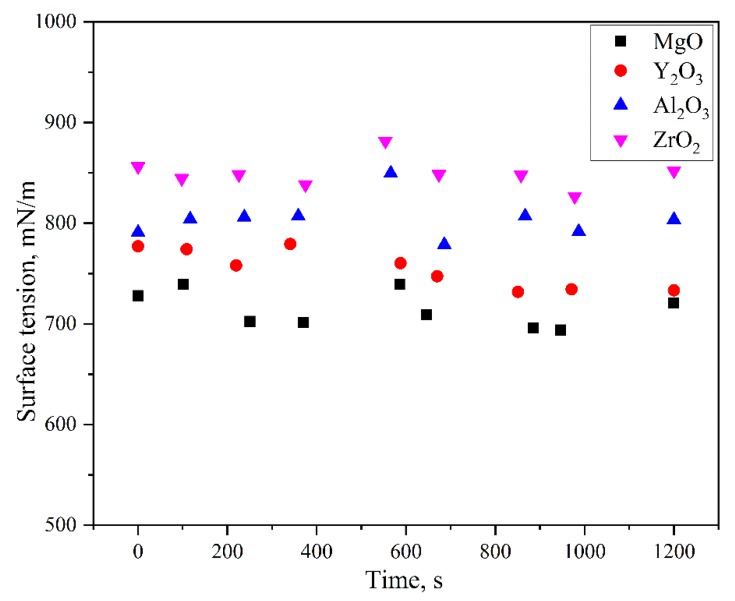
Variation in the surface tension with time with regard to the wetting of the molten alloys on the different substrates at 1873 K.

The variation of the surface tension as a function of time on the different substrates is shown in Figure 10. The surface tension in the present work was much higher than 670–700 mN/m. And the average surface tension with respect to the type of the ceramic substrates gradually increased, ZrO_2_ > Al_2_O_3_ > Y_2_O_3_ > MgO.

**Figure 11:**
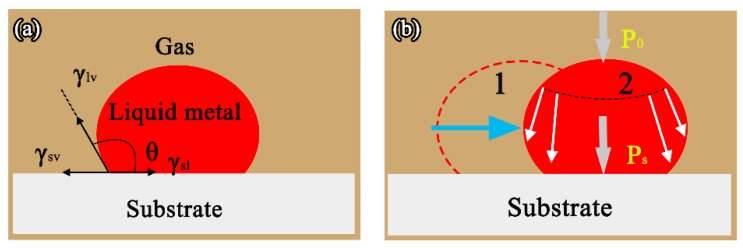
Schematic configurations of a sessile drop in the case of θ > 90°: (**a**) at the solid-liquid-vapor triple line; and (**b**) from 1st to 2nd position.

A schematic representation of the molten alloys from the 1st to 2nd position is shown in Figure 11b. Due to the surface tension (Po), the liquid at the curved surface was different from the plane and the liquid molecules at the surface were always subjected to an additional contraction pressure (Ps), pointing to the center of the sphere. Under the action of P (P = Po + Ps), the melt alloys had a non-wetting displacement without spreading on the crucible wall, as shown in Figure 11.

**Figure 12:**
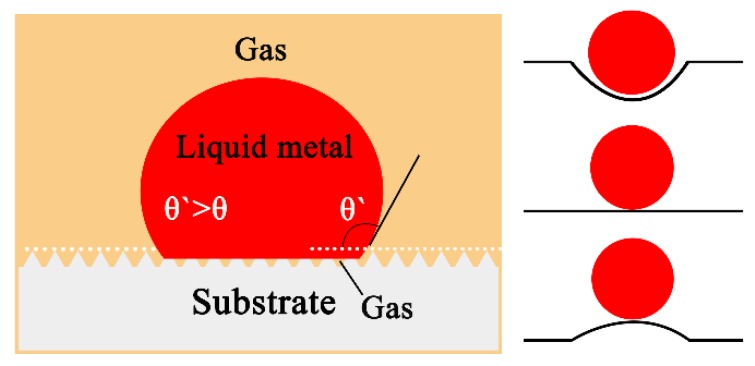
Schematic configurations of a sessile drop resting on the substrate with rough surfaces in the case of θ > 90°.

The influence of substrate surface roughness on the wetting behavior could be attributed to the pinning of the triple line by sharp edges that act as obstacles to the spreading of the liquid [27]. According to Wenzel [28] and Cassie and Baxter [29], in the region of wettability (θ < 90°), roughness must cause the contact angle to decrease in comparison with the contact angle on a smooth surface. In the system of non-wettability (θ > 90°), the equilibrium angle on the rough substrate surface would be higher, that is, θ’ > θ. The rough interface reduced contact area between the molten alloys and substrate as shown in Figure 12.

**Figure 13:**
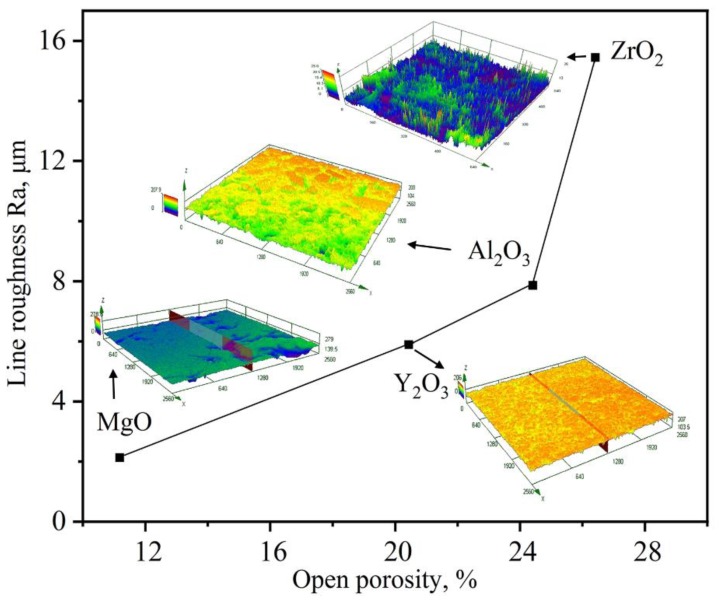
The relationship between the surface roughness values (Ra) and the levels of open porosity of the various oxide ceramics.

In this study, in any case, the contact angle of the alloy/ceramic system was larger than 140° within the measuring time. The relationship between the surface line roughness values and the levels of open porosity of the ceramic substrates is shown in Figure 13. There was a positive correlation between the surface roughness values and the levels of open porosity. According to the experimental results in Figure 13, in the case of the substrates with open porosity levels of 11.8% (MgO), 20.44% (Y_2_O_3_), 24.41% (Al_2_O_3_), and 26.42% (ZrO_2_), the surface roughness values, Ra, were determined to be 2.127 μm (MgO), 5.886 μm (Y_2_O_3_), 7.856 μm (Al_2_O_3_), and 15.44 μm (ZrO_2_), respectively. The surface roughness of the four different oxide substrates gradually increased and these surface structures were more complicated. The pinning of the triple line by any sharp edges could also obstruct the spreading of the liquid. Thus, the contact angle would increase as the surface roughness increased.

### 4.2. Thermodynamic Analysis of Interfacial Reactions

In the four non-wetting systems, Y segregated at the interface, and then precipitated as a reaction product, Y_2_O_3_. According to the Gibbs free energy of formation [30], shown in Table 4, the thermodynamic stability of the refractory can be described by the following sequence: Y_2_O_3_ > ZrO_2_ > Al_2_O_3_ > MgO. Saha proposed that, when there was little variation in the free energy, the contamination of the alloy matrix owed to refractory oxides may not just be related to the thermodynamic stability of the oxide, but may also be influenced by the solution effect [31]. Therefore, the occurrence of the interfacial reactions in the alloy/ceramic substrate could be ascribed to the thermal dissociation of MgO, Al_2_O_3_, and ZrO_2_, which was shown in Table 4:

In the three systems, sufficient [O] atoms were dissolved during the reversible reaction, and the Y tended to segregate at the alloy/ceramic interface. Thus, the released [O] atoms could combine with Y to form Y_2_O_3_. The melting point of Y_2_O_3_ (1873 K) was greater than that of MgO, Al_2_O_3_, and ZrO_2_ (1873 K), and the value of free energy change associated with reactions (3), (4), and (5) should be lower than that of reaction (6). Therefore, the Y_2_O_3_ reaction layer should precipitate (reaction (6)) at the interface. When the diffusion in the molten alloys was more rapid than the interfacial reaction, the spreading was limited by the interfacial reaction. However, if the growth of the reaction product at the triple line was limited by the transmission of reaction elements, the spreading was limited by diffusion [32].

In the case of the MgO system, the fine MgO particles were sintered together, which indicated that thermal dissociation occurred at the interface and within the substrate. When the [O] atoms at the interface were consumed by Y to form Y_2_O_3_, the [O] atoms located far from the interface could easily travel to the interface because of the proliferation of the molecules at high temperature. However, a reaction layer had formed at the interface. Therefore, the new [O] atoms would diffuse into the molten alloys owing to the chemical potential gradient of [O] between the MgO substrate and the molten alloys. When the [O] content reached a certain limit, oxide inclusions would precipitate within the alloy matrix.

In the other two systems, owing to the low degree of sintering and the presence of the raw, inactive Al_2_O_3_ and ZrO_2_ particles at the surface, compared with the MgO substrate, there were fewer [O] atoms released. When the molten alloys were dropped, there were insufficient [O] atoms present to react with the Y to form Y_2_O_3_ immediately. Therefore, the reaction layer was irregular compared to that of the MgO system. When the [O] atoms located far from the interface moved to the interface, the formed reaction layer prevented contact between the Y and [O]. Therefore, the reaction layer could not increase, and fewer [O] atoms would diffuse into the alloys. It is noteworthy that the Al_2_O_3_ and ZrO_2_ system possessed the lower oxide inclusion content. This could be attributed to the surface structure. In the case of the MgO system, the MgO substrate with lower thermal stability should surely lead to high oxide inclusions. In the case of the Al_2_O_3_ and ZrO_2_ systems, substrates with higher thermal stability and low degree of sintering resulted in a weak reaction. Moreover, in the case of the Al_2_O_3_ system, Y_2_O_3_ and Al_2_O_3_ had the same lattice structure, and they could form a continuous solid solution at a certain temperature. A thin layer of Y_2_O_3_ solid solution between the reaction layer and the sand adhesion layer formed.

Based on the analysis above, the wetting behavior and interactions between Ni-20Co-20Cr-10Al-1.5Y alloys and various oxide ceramics were affected by the existence of the Y_2_O_3_ layer at the interface. The alloys after reacting with the MgO ceramic had the highest inclusion content, while those of the lowest content were in the Y_2_O_3_ system. This indicated that Y_2_O_3_ ceramic was most beneficial with regard to the vacuum induction melting of high-purity Ni-20Co-20Cr-10Al-1.5Y alloys.

## 5. Conclusions

The wetting of molten Ni-20Co-20Cr-10Al-1.5Y alloys on MgO, Y_2_O_3_, Al_2_O_3_, and ZrO_2_ ceramic substrates were studied by an improved sessile-drop method at 1873 K. Based on which the high-temperature wettability, interactions, and associated mechanisms were discussed. The primary conclusions of this work are as follows:

- With an increase of the experiment time, the contact angles of molten alloys on MgO, Y_2_O_3_, Al_2_O_3_, and ZrO_2_ substrates rapidly became stable. The equilibrium angles were 140°, 148°, 154°, and 157°, respectively. There was no characteristic transition in wettability when the type of substrates changed and no alloy liquid penetrated into the substrates. The influence of different ceramics on the wettability can be explained by the surface tension of the melts and the surface roughness of the substrates.
- With the exception of the Y_2_O_3_ system, an approximately 25 μm continuous Y_2_O_3_ reaction layer was observed along the interface of the alloys. The Y_2_O_3_ layer significantly reduced the wettability and interactions of Ni-20Co-20Cr-10Al-1.5Y alloys on MgO, Al_2_O_3_, and ZrO_2_.
- Oxide particles were found in the alloy matrices of all the systems. The average area percentage of oxides were 0.59% (MgO), 0.11% (Al_2_O_3_), 0.09% (ZrO_2_), and 0.02% (Y_2_O_3_), respectively. The formation of oxides could be attributed to the thermal dissociation of substrates, which provided sufficient [O] atoms that could dissolve into the molten alloys easily.
- The Y_2_O_3_ ceramic with open porosity levels of 20.44% was most beneficial with regard to the vacuum induction melting of high-purity Ni-20Co-20Cr-10Al-1.5Y alloys.

## Figures and Tables

**Table 1:** Parameters related to substrates with various oxide ceramics.

Parameters	MgO	Y_2_O_3_	Al_2_O_3_	ZrO_2_
Purity (%)	99.9	99.9	99.9	94
Particle size (mesh)	500	325	200–300	200
Grinding time (min)	10	10	10	10
PVA binder (wt %)	5	5	5	5
Molding pressure (MPa)	10	10	10	10
Open porosity (%)	11.8	20.44	24.41	26.42

**Table 2:** Chemical compositions obtained via EDS analysis (at%).

Element	O	Al	Y	Cr	Co	Ni	Zr
Point 1	8.52	23.68	0.45	9.51	11.74	46.10	-
Point 2	2.29	37.71	0.35	9.19	10.88	39.58	-
Point 3	2.44	7.05	0.21	26.74	21.38	42.17	-
Point 4	12.60	5.67	2.76	14.31	11.99	30.57	22.10

**Table 3:** Oxide inclusion contents of the alloys, based on the images of the longitudinal sections of the alloys (excluding the reaction layer).

Systems	Number of Oxide Particles	Average Diameter of Oxide Particles/μm^3^	Area Percentage of Oxide Inclusion
MgO	57	31.3	0.59%
Al_2_O_3_	125	6.8	0.11%
ZrO_2_	64	10.1	0.09%
Y_2_O_3_	42	3.9	0.02%

**Table 4:** Gibbs free energy of formation of the metallic oxides at 1900 K.

Oxides	ΔG_f_ (kJ/mol)	No.
MgO(s) = Mg(l) + O(g)	339.741	(3)
1/3Al_2_O_3_(s) = 2/3Al(l) + O(g)	355.326	(4)
1/2ZrO_2_(s) = 1/2Zr(l) + O(g)	372.979	(5)
1/3Y_2_O_3_(s) = 2/3Y(l) + O(g)	454.243	(6)
Al_2_O_3_(s) ↔ Al_2_O(g) + O(g)	-	(7)
